# Survival benefits of Cytoreductive Nephrectomy in patients with metastatic renal cell carcinoma: evidence from a SEER-based retrospective cohort study

**DOI:** 10.1371/journal.pone.0318896

**Published:** 2025-07-15

**Authors:** Xiongwu Peng, Lingxing Duan, Qi Wu, Shiping Wu, Wenfeng Wang, Runlin Shi

**Affiliations:** 1 Department of Urology, Nanchang University Second Affiliated Hospital, Nanchang, Jiangxi Province, China; 2 Department of Urology, Pingxiang Third People’s Hospital, Pingxiang, Jiangxi Province, China; 3 Department of Cardiology, Wuhan Asia General Hospital, Wuhan, Hubei Province, China; Memorial Sloan Kettering Cancer Center, UNITED STATES OF AMERICA

## Abstract

**Background:**

Metastatic renal cell carcinoma (mRCC) is associated with poor prognosis, with a 5-year survival rate of less than 15%. Cytoreductive nephrectomy (CN) has historically played a critical role in mRCC management, potentially enhancing systemic therapy efficacy by reducing tumor burden. However, its relevance in the era of targeted therapies and immune checkpoint inhibitors (ICIs) has been questioned.

**Objective:**

This study evaluates the survival benefits of CN in mRCC patients using real-world, population-based data from the SEER database.

**Methods:**

A retrospective cohort analysis of 6,030 mRCC patients was performed using data from 2010 to 2017. Propensity score matching (PSM) minimized selection bias, yielding 1,350 matched patients. Kaplan-Meier survival curves and multivariate Cox proportional hazards models assessed the impact of CN on overall survival (OS) and RCC-specific survival (CSS), stratified by demographic and clinical characteristics.

**Results:**

CN was associated with a 71% reduction in all-cause mortality (HR = 0.29, 95% CI = 0.25–0.33) and RCC-specific mortality (HR = 0.29, 95% CI = 0.25–0.34). Five-year OS rates were 31.5% in the CN group versus 3.6% in the non-CN group. Survival benefits were consistent across subgroups, including patients with high-grade or advanced-stage tumors, underscoring the role of CN within multimodal treatment strategies.

**Conclusion:**

CN confers significant survival advantages in mRCC, even in challenging clinical scenarios. These findings reinforce the importance of integrating CN into multimodal therapeutic frameworks, particularly alongside modern systemic therapies. Further prospective studies are warranted to optimize patient selection and treatment sequencing.

## Introduction

Renal cell carcinoma (RCC) accounts for 3%–5% of global malignancies [1]. It represents the most prevalent form of kidney cancer, representing nearly 90% of all renal neoplasms [2]. Despite advancements in early detection and therapeutic interventions, a substantial proportion of patients are diagnosed with metastatic disease (M1 stage), which is linked to a five-year survival rate of under 15% [3,4]. These statistics underscore the pressing need to develop more effective treatment strategies to improve outcomes for this vulnerable patient population.

Cytoreductive nephrectomy (CN), a surgical intervention targeting the removal of the primary tumor, has traditionally been a key component in the treatment of metastatic renal cell carcinoma (mRCC) [5]. The procedure is hypothesized to alleviate tumor burden, potentiate the host immune response, and augment the efficacy of subsequent systemic therapies [6]. However, with the rise of targeted therapies like tyrosine kinase inhibitors (TKIs) and immune checkpoint inhibitors (ICIs), the significance of CN has come under growing scrutiny [7–9]. Landmark randomized trials, including CARMENA and SURTIME, have cast doubt on the universal benefit of CN, particularly in patients with poor performance status or extensive metastatic burden [8–12].

The CARMENA trial, which evaluated the efficacy of CN followed by sunitinib versus sunitinib monotherapy in patients with metastatic renal cell carcinoma (mRCC), revealed that the addition of CN did not significantly enhance overall survival. This result calls into question the long-held assumption that CN is indispensable for all individuals with metastatic disease. Conversely, the SURTIME trial, which explored the timing of CN, demonstrated that performing CN after the initiation of systemic therapy (with sunitinib) conferred a survival advantage over immediate CN followed by sunitinib. These studies have provoked critical reflections on patient selection for CN, suggesting that its benefits may not be universally applicable, especially in patients with poor prognostic factors. Nevertheless, the applicability of these findings to real-world populations remains constrained by the trials’ restrictive inclusion criteria and limited sample sizes [13]. Consequently, the optimal patient subgroups most likely to derive survival benefit from CN in the modern era of systemic therapies remain inadequately defined [8].

Given these uncertainties, we postulate that CN retains the potential to confer substantial survival benefits in carefully selected patients with mRCC, especially when integrated into a multimodal therapeutic framework. To test this hypothesis, we analyzed data from the Surveillance, Epidemiology, and End Results (SEER) database, a comprehensive population-based cancer registry. Our study design entailed meticulous patient selection, extensive stratification, and rigorous statistical analyses to evaluate the impact of CN on overall survival (OS) and RCC-specific survival (RCC-SS).

In addition, this investigation employed stratified analyses across sociodemographic, clinical, and pathological characteristics to rigorously evaluate the potential survival advantages conferred by CN. Kaplan-Meier survival curves and Cox proportional hazards models revealed significantly lower overall and RCC-specific mortality among patients undergoing CN compared to those receiving non-surgical management. Furthermore, stratified analyses demonstrated a remarkable consistency in survival benefits across diverse clinical subgroups, underscoring the integral role of CN within multimodal treatment paradigms.

By employing a propensity score-matched cohort and leveraging advanced statistical methodologies, including Cox proportional hazards and Fine and Gray competing risks models, we identified critical patient subgroups most likely to benefit from CN. This research provides important insights into the current role of CN in clinical practice, helping to refine therapeutic approaches for patients with mRCC. Ultimately, our findings emphasize the imperative to identify patient-specific factors to maximize the therapeutic efficacy of CN and improve long-term outcomes in this challenging disease landscape.

## Methods

### Data source

The SEER database, utilized in this analysis, includes data from 17 U.S. states between 2000 and 2019, as per its 2021 edition. Covering approximately 26.5% of the U.S. population, it provides a comprehensive resource for examining cancer incidence, survival, and treatment patterns. Its geographic and demographic diversity, coupled with rigorous data standards, ensures robust insights into cancer trends and disparities.

The Ethics Committee of the Third People’s Hospital of Pingxiang City granted an exemption for this study, as the SEER program solely utilizes anonymized, population-based cancer registry data. Since the database is publicly available and does not require direct engagement with human subjects, formal ethical approval was considered redundant.

### Cohort selection

The case list was generated using SEER*Stat software, version 8.4.4, and included patients diagnosed with metastatic (M1 stage) renal parenchymal carcinoma, classified under ICD-O code C64.9. The dataset captured an extensive array of variables, encompassing demographic factors (age, sex, ethnicity, marital status), clinical characteristics (year of diagnosis, tumor grade, laterality, histologic subtype, T and N stages), and treatment details (radiation therapy and chemotherapy). This comprehensive dataset forms a robust foundation for analyzing disease patterns, treatment strategies, and outcomes in metastatic renal cell carcinoma.

To refine the cohort, 124,007 cases diagnosed between 2000–2009 and 2018–2019 were excluded, restricting the analysis to diagnoses made between 2010 and 2017. Patients under the age of 18 were further excluded, resulting in an initial cohort of 91,842 patients. Subsequently, cases with missing T and N staging, patients who had received chemotherapy or radiotherapy, and those who had not undergone partial nephrectomy (PN) or radical nephrectomy (RN) were excluded, eliminating 7,610 cases. Additionally, cases with incomplete outcome data and those diagnosed with renal pelvis cancer (International Classification of Diseases for Oncology code C65.9) were removed, further reducing the cohort by 96 cases. This rigorous selection process yielded a final cohort of 6,030 eligible patients.

To address potential selection bias, propensity score matching (PSM) was employed, resulting in a matched cohort of 1,350 patients for subsequent analysis.

### Vital status

Patient status at the latest follow-up was assessed using the SEER9 “cause of death (COD) to site recode” variable, allowing precise classification of survival outcomes. Patients were categorized as alive at the most recent follow-up, deceased due to renal cell carcinoma, or deceased from non-renal causes. The primary endpoint of the analysis was overall mortality, providing a comprehensive measure of survival. Secondary outcomes included renal cancer-specific mortality and deaths attributable to non-renal causes, offering critical insights into the interplay of disease progression and competing health risks.

Temporal data were derived from the “months of survival” variable, which spanned from the date of diagnosis to the most recent follow-up. Survival duration, expressed in months, was computed using SEER*Stat software by subtracting the diagnosis date from the last contact date (or study endpoint). The calculation employed a conversion factor of 365.24 days per year divided by 12 to approximate the number of days in a month. The study endpoint was set as December 31, 2019.

### Balancing cohorts using propensity score matching

Propensity scores were calculated using logistic regression, incorporating variables such as demographics (age, sex, race), tumor characteristics (T stage, N stage, laterality, histologic subtype, grade), sequence number, and tumor count to adjust for treatment selection bias. A one-to-one matched cohort was then created using the nearest-neighbor method with a caliper width of 0.001, ensuring precise pairing and balance between surgical and non-surgical groups for outcome comparison.

### Statistical analysis

Descriptive analyses were conducted for all variables, with patients categorized according to the treatment modality they received, classified as either surgical or non-surgical. Non-surgical management was defined as the absence of surgery, while surgical intervention included only PN or RN. OS was defined as the period from the date of RCC diagnosis to death from any cause, while RCC-SS was the duration from diagnosis to death specifically due to RCC.

Univariate (unadjusted) analyses were performed to identify covariates associated with mortality, and stratified (adjusted) analyses were employed to evaluate the differential impact of treatment within specific population subgroups. KM curves were used to construct survival curves for patients with RCC, stratified by treatment type. KM curves for all-cause mortality and RCC-SS, further stratified by T and N stages, were generated to examine treatment effects across various subgroups.

The Cox proportional hazards model was employed to evaluate the influence of various factors, including age, tumor laterality, race, tumor grade, year of diagnosis, histologic subtype, sex, T and N stages, treatment modality, sequence number, tumor burden, and marital status, on both overall mortality and RCC-SS. Furthermore, the cumulative incidence of competing risks was utilized to estimate RCC-specific mortality, while appropriately accounting for the impact of competing events.

All statistical analyses were conducted using Empower (R) (www.empowerstats.com, X&Y Solutions, Inc., Boston, MA, USA) and R version 3.6.3 (http://www.R-project.org), both of which are recognized for their robust data processing capabilities and extensive analytical features. Statistical significance was considered achieved when the p-value was less than 0.05.

## Results

### Baseline characteristics of the patient population

This is shown in Table 1, of the entire cohort, only 1,560 patients (25.9%) underwent surgical resection of the primary tumor, whereas the majority (4,471 patients, 74.1%) did not receive any surgical intervention. Patients who underwent surgery were disproportionately older, white, married, and diagnosed with clear cell carcinoma, suggesting that both demographic characteristics and tumor-specific features exerted considerable influence over treatment decisions. Moreover, the choice between surgical and non-surgical management was strongly associated with tumor stage and grade. In particular, among patients with T3 stage tumors, only 16.09% did not undergo surgical resection, underscoring the pivotal role of surgical intervention in the management of advanced-stage disease.

**Table 1:** Baseline Characteristics of the Patient Population.

	Original cohort			Matching cohort		
	No surgery	Surgery	P-value	No surgery	Surgery	P-value
Age			<0.001			0.821
20-29	1 (0.02%)	7 (0.45%)		1 (0.14%)	2 (0.31%)	
30-39	28 (0.63%)	26 (1.67%)		13 (1.87%)	8 (1.22%)	
40-49	171 (3.83%)	155 (9.94%)		49 (7.05%)	46 (7.02%)	
50-59	647 (14.47%)	398 (25.51%)		148 (21.29%)	134 (20.46%)	
60-69	1153 (25.79%)	510 (32.69%)		223 (32.09%)	230 (35.11%)	
70-79	1163 (26.02%)	351 (22.50%)		175 (25.18%)	163 (24.89%)	
80+	1307 (29.24%)	113 (7.24%)		86 (12.37%)	72 (10.99%)	
Sex			0.004			0.027
Male	2918 (65.28%)	1081 (69.29%)		478 (68.78%)	413 (63.05%)	
Female	1552 (34.72%)	479 (30.71%)		217 (31.22%)	242 (36.95%)	
Year of diagnosis			0.223			0.699
2010-2013	2143 (47.94%)	720 (46.15%)		315 (45.32%)	290 (44.27%)	
2014-2017	2327 (52.06%)	840 (53.85%)		380 (54.68%)	365 (55.73%)	
Race			<0.001			0.440
White	3679 (82.30%)	1313 (84.17%)		560 (80.58%)	541 (82.60%)	
Black	509 (11.39%)	116 (7.44%)		76 (10.94%)	58 (8.85%)	
Other	282 (6.31%)	131 (8.40%)		59 (8.49%)	56 (8.55%)	
Marital status			<0.001			0.458
Married	2035 (45.53%)	970 (62.18%)		371 (53.88%)	366 (55.88%)	
Widowed	842 (18.84%)	122 (7.82%)		80 (11.51%)	63 (9.62%)	
Other	1593 (35.64%)	468 (30.00%)		244 (35.11%)	226 (34.50%)	
Grade			<0.001			<0.001
G1	64 (1.43%)	33 (2.12%)		41 (5.90%)	12 (1.83%)	
G2	164 (3.67%)	281 (18.01%)		114 (16.40%)	85 (12.98%)	
G3	300 (6.71%)	544 (34.87%)		139 (20.00%)	184 (28.09%)	
G4	94 (2.10%)	471 (30.19%)		43 (6.19%)	178 (27.18%)	
G_X_	3848 (86.09%)	231 (14.81%)		358 (51.51%)	196 (29.92%)	
Laterality			<0.001			<0.001
Left – origin of primary	2031 (45.44%)	829 (53.14%)		321 (46.19%)	341 (52.06%)	
Right – origin of primary	2054 (45.95%)	725 (46.47%)		347 (49.93%)	309 (47.18%)	
Unknown	385 (8.61%)	6 (0.38%)		27 (3.88%)	5 (0.76%)	
Histologic type			<0.001			0.530
Clear cell carciinoma	999 (22.35%)	1067 (68.40%)		368 (52.95%)	358 (54.66%)	
Papillary cell carcinoma	131(2.93%)	93(5.96%)		24(3.45%)	52(7.94%)	
Chromophobe renal carcinoma	20(0.45%)	18(1.15%)		2(0.29%)	12(1.83%)	
Non-Clear cell carcinoma	3471 (77.65%)	493 (31.60%)		300 (43.17%)	235 (35.88%)	
T			<0.001			<0.001
T1	1088 (24.34%)	233 (14.94%)		156 (22.45%)	109 (16.64%)	
T2	777 (17.38%)	219 (14.04%)		137 (19.71%)	106 (16.18%)	
T3	719 (16.09%)	921 (59.04%)		182 (26.19%)	334 (50. 99%)	
T4	471 (10.54%)	166 (10.64%)		90 (12.95%)	90 (13.74%)	
T_X_	1415 (31.66%)	21 (1.35%)		130 (18.71%)	16 (2.44%)	
N			<0.001			0.062
N0	2217 (49.60%)	1139 (73.01%)		427 (61.44%)	426 (65.04%)	
N1	1228 (27.47%)	349 (22.37%)		184 (26.47%)	175 (26.72%)	
N_X_	1025 (22.93%)	72 (4.62%)		84 (12.09%)	54 (8.24%)	
Sequence number			<0.001			0.764
First/only primary	3607 (80.69%)	1343 (86.09%)		574 (82.59%)	545 (83.21%)	
Second/higher-order primary	863 (19.31%)	217 (13.91%)		121 (17.41%)	110 (16.79%)	
Number of tumors			0.057			0.883
1	3473 (77.70%)	1253 (80.32%)		543 (78.13%)	511 (78.02%)	
2	791 (17.70%)	252 (16.15%)		120 (17.27%)	117 (17.86%)	
3+	206 (4.61%)	55 (3.53%)		32 (4.60%)	27 (4.12%)	

**Fig 1:**
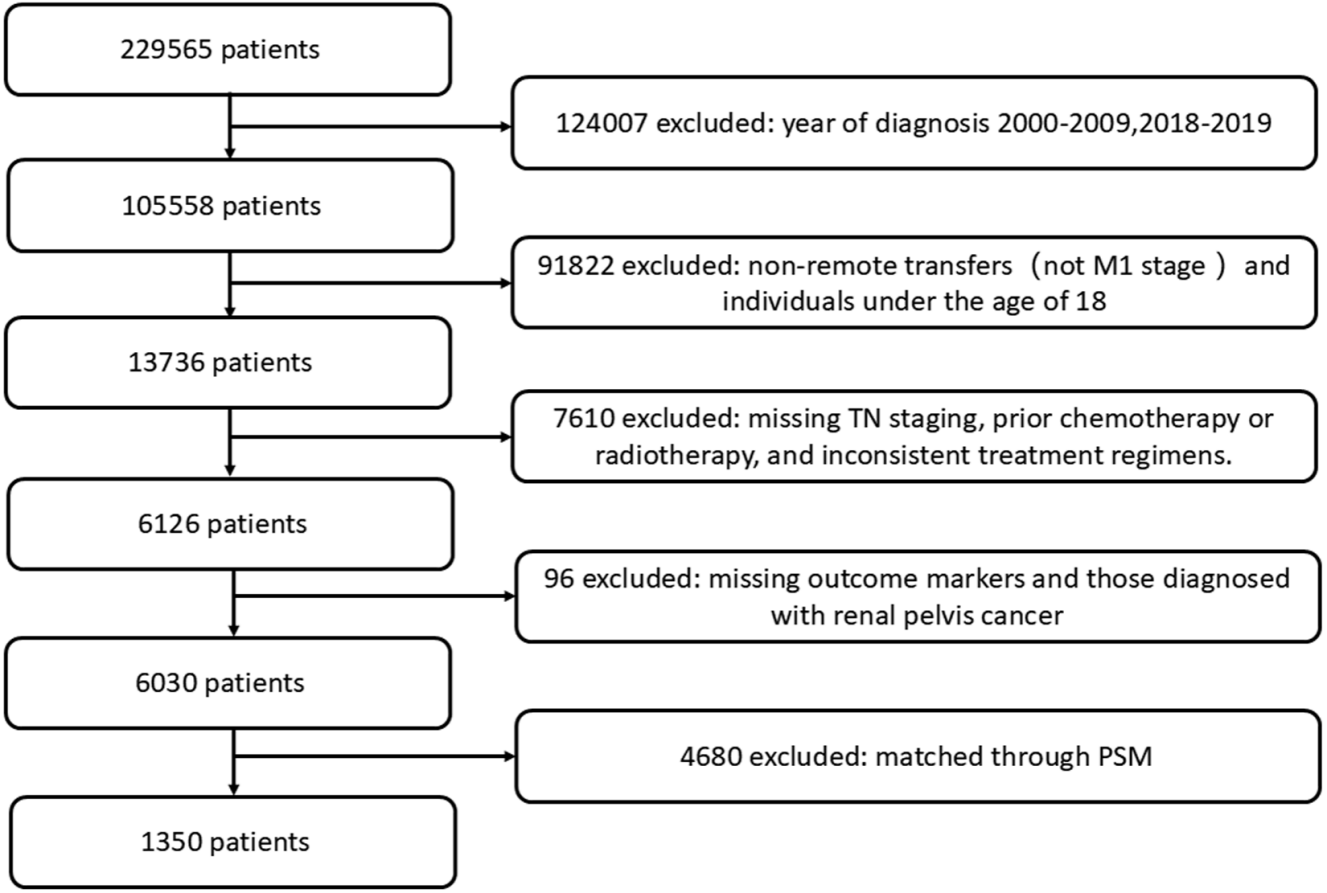
Flow chart used to select participants.

As shown in Fig 1, to minimize potential confounding factors and enable robust comparisons, a propensity score-matched cohort comprising 1,350 patients was established. Of these, 655 individuals were allocated to the surgical group, while 695 were assigned to the non-surgical group. Following the matching process, the baseline clinical characteristics between the two cohorts were largely comparable, with no significant differences observed, except for a few variables such as sex, tumor grade, laterality, and T stage. This methodologically rigorous approach substantially mitigated bias, thereby enabling a more reliable and nuanced evaluation of treatment outcomes between the surgical and non-surgical cohorts.

Surgery, the patients underwent cytoreductive nephrectomy; no surgery, the patients did not undergo cytoreductive nephrectomy. Continuous variables are presented as the mean± SD; Categorical variables are presented as n (%).

### Univariate analysis

As shown in Table 2, the unadjusted analysis revealed a significant survival advantage for the surgical cohort, with lower all-cause mortality (hazard ratio [HR] = 0.40) and renal cell carcinoma-specific mortality (HR = 0.30) compared to those in the non-surgical group. These results were highly statistically significant (p < 0.0001), suggesting that surgical resection is associated with improved overall survival and RCC-specific survival.

**Table 2:** Univariate analysis.

Exposure	Statistics	Hazard Ratio(95%CI) p-value	
		Kidney cancer-specific mortality	All-cause mortality
Age			
20-29	3 (0.22%)	1.0	1.0
30-39	21 (1.56%)	0.73 (0.21, 2.51) 0.6187	0.76 (0.22, 2.60) 0.6657
40-49	95 (7.04%)	0.61 (0.19, 1.95) 0.4081	0.65 (0.20, 2.06) 0.4630
50-59	282 (20.89%)	0.63 (0.20, 1.98) 0.4304	0.67 (0.22, 2.10) 0.4953
60-69	453 (33.56%)	0.66 (0.21, 2.07) 0.4778	0.72 (0.23, 2.24) 0.5691
70-79	338 (25.04%)	0.67 (0.21, 2.10) 0.4939	0.77 (0.25, 2.40) 0.6485
80+	158 (11.70%)	0.82 (0.26, 2.59) 0.7407	1.04 (0.33, 3.28) 0.9418
Sex			
Male	891 (66.00%)	1.0	1.0
Female	459 (34.00%)	0.97 (0.85, 1.11) 0.6664	0.97 (0.86, 1.10) 0.6750
Year of diagnosis			
2010-2013	605 (44.81%)	1.0	1.0
2014-2017	745 (55.19%)	0.87 (0.77, 0.99) 0.0391	0.90 (0.80, 1.02) 0.0961
Race			
White	1101 (81.56%)	1.0	1.0
Black	134 (9.93%)	1.18 (0.96, 1.45) 0.1156	1.19 (0.98, 1.45) 0.0732
Other	115 (8.52%)	0.98 (0.78, 1.24) 0.8846	0.97 (0.78, 1.20) 0.7765
Marital status			
Married	737 (54.59%)	1.0	1.0
Widowed	143 (10.59%)	1.40 (1.14, 1.72) 0.0013	1.41 (1.16, 1.71) 0.0005
Other	470 (34.81%)	1.03 (0.90, 1.18) 0.6913	1.02 (0.90, 1.16) 0.7249
Grade			
G1	53 (3.93%)	1.0	1.0
G2	199 (14.74%)	0.69 (0.48, 1.00) 0.0482	0.69 (0.49, 0.96) 0.0289
G3	323 (23.93%)	0.97 (0.69, 1.37) 0.8600	0.94 (0.69, 1.29) 0.6948
G4	221 (16.37%)	1.18 (0.83, 1.68) 0.3443	1.02 (0.74, 1.41) 0.9088
GX	554 (41.04%)	1.08 (0.78, 1.51) 0.6388	1.02 (0.75, 1.38) 0.9154
Laterality			
Left – origin of primary	662 (49.04%)	1.0	1.0
Right – origin of primary	656 (48.59%)	1.03 (0.90, 1.17) 0.6823	1.00 (0.88, 1.12) 0.9433
3	32 (2.37%)	1.08 (0.71, 1.64) 0.7262	1.21 (0.84, 1.75) 0.3015
Histologic type			
Clear cell carcinoma	725 (53.78%)	1.0	1.0
Palliary cell carcinoma	76(5.63%)	1.09(0.82,1.44)0.5643	1.13(0.87,1.46)0.3756
Chromophobe cell carcinoma	14(1.04%)	0.51(0.23,1.14)0.1019	0.52(0.25,1.10)0.0880
Non-Clear cell	535 (39.63%)	1.58 (1.38, 1.80) <0.0001	1.59 (1.40, 1.80) <0.0001
T			
T1	265 (19.63%)	1.0	1.0
T2	243 (18.00%)	1.34 (1.07, 1.68) 0.0102	1.12 (0.91, 1.37) 0.2854
T3	516 (38.22%)	1.40 (1.16, 1.70) 0.0006	1.17 (0.98, 1.39) 0.0779
T4	180 (13.33%)	2.42 (1.93, 3.05) <0.0001	1.97 (1.59, 2.42) <0.0001
TX	146 (10.81%)	1.92 (1.50, 2.46) <0.0001	1.68 (1.34, 2.10) <0.0001
N			
N0	853 (63.19%)	1.0	1.0
N1	359 (26.59%)	1.79 (1.55, 2.07) <0.0001	1.73 (1.51, 1.98) <0.0001
NX	138 (10.22%)	1.27 (1.03, 1.57) 0.0282	1.26 (1.03, 1.54) 0.0218
Treatment			
No surgery	695 (51.48%)	1.0	1.0
Surgery	655 (48.52%)	0.37 (0.32, 0.42) <0.0001	0.36 (0.32, 0.41) <0.0001
Sequence number			
First/only primary	1119 (82.89%)	1.0	1.0
Second/higher-order primary	231 (17.11%)	0.80 (0.67, 0.95) 0.0120	0.94 (0.80, 1.10) 0.4141
Number of tumors			
1	1054 (78.07%)	1.0	1.0
2	237 (17.56%)	0.78 (0.66, 0.93) 0.0065	0.92 (0.78, 1.07) 0.2765
3+	59 (4.37%)	0.61 (0.42, 0.88) 0.0085	0.90 (0.67, 1.21) 0.4868

Further investigation into the factors influencing survival outcomes highlighted the complexity of the disease, as both all-cause and RCC-SS were influenced by multiple clinical and demographic variables, including marital status, tumor grade, histologic subtype, T stage, and N stage. The multivariate regression analysis, adjusted for these factors, reinforced the survival benefits of surgery across different patient subgroups.

### Stratified analysis

The stratified adjusted analysis (Supplementary S1 Table) provides a detailed examination of the influence of CN on survival outcomes in mRCC patients, taking into account variables such as age, sex, race, tumor grade, histologic subtype, and staging. The results indicate that patients who underwent CN exhibited a significant reduction in both all-cause mortality and kidney cancer-specific mortality across various subgroups.

Notably, younger patients (40–49 years: HR = 0.34, p < 0.0001), both males (HR = 0.38, p < 0.0001) and females (HR = 0.35, p < 0.0001), White individuals (HR = 0.35, p < 0.0001), and those with clear cell histology (HR = 0.33, p < 0.0001) derived the most pronounced survival benefits. Patients with lower-stage tumors (T1–T3) and lower-grade lesions (G1–G2) exhibited the greatest advantage (e.g., G2: HR = 0.17, p < 0.0001). Although patients with advanced-stage (T4) or high-grade tumors demonstrated comparatively smaller benefits, the survival advantage conferred by CN remained statistically significant.

Collectively, these results underscore the pivotal role of CN within multimodal therapeutic strategies, highlighting its substantial survival benefits in specific, well-defined patient subgroups.

### Multivariate analysis

In our multivariate regression analysis, patients who underwent surgical resection demonstrated substantially lower rates of both OS and RCC-SS compared to their non-surgical counterparts (Table 3). When evaluating OS, the surgical cohort exhibited a markedly reduced risk of mortality across all models, including the unadjusted analysis and Models I and II, which incorporated adjustments for sociodemographic and clinical variables. Notably, the hazard ratio (HR) for OS among patients who underwent surgery was 0.37 (95% CI = 0.37–0.42, p < 0.0001), corresponding to a 63% reduction in mortality risk. This survival benefit remained statistically robust throughout all analytical frameworks, underscoring the pivotal role of surgical intervention in enhancing long-term outcomes.

**Table 3:** Multivariate analysis.

	Hazard Ratio (95% CI) p-value		
Exposure	Non-adjusted	Adjust I	Adjust II
Cause			
Treatment			
No surgery	1.0	1.0	1.0
Surgery	0.37 (0.32, 0.42) <0.0001	0.37 (0.32, 0.42) <0.0001	0.29 (0.25, 0.33) <0.0001
Vital status			
Treatment			
No surgery	1.0	1.0	1.0
Surgery	0.36 (0.32, 0.41) <0.0001	0.36 (0.32, 0.41) <0.0001	0.29 (0.25, 0.34) <0.0001

Similarly, the analysis of RCC-specific survival highlighted a consistent and significant benefit for patients who underwent surgical resection. Across both unadjusted and adjusted models, the risk of RCC-SS was reduced by 64% in the surgical cohort (HR = 0.36, 95% CI = 0.32–0.41, p < 0.0001). This benefit was even more pronounced in the fully adjusted model, which accounted for an extensive array of sociodemographic and clinical confounders, yielding an HR of 0.29 (95% CI = 0.25–0.34, p < 0.0001). This represents a remarkable 71% reduction in RCC-specific mortality, further emphasizing the significant protective effect of surgical treatment.

### OS and RCC-SS across treatment modalities

Patients who underwent surgical intervention demonstrated markedly superior survival outcomes compared to those who did not undergo surgery (Table 4). For overall survival (OS), the 1-year, 3-year, and 5-year survival rates were 60.97%, 41.79%, and 31.50%, respectively, in the surgery group, as opposed to 23.96%, 8.05%, and 3.56% in the non-surgery group. Similarly, RCC-SS at 1, 3, and 5 years was 63.87%, 46.23%, and 36.20% for the surgery group, compared to 28.40%, 11.47%, and 5.77% for the non-surgery group. These findings underscore the significant survival advantage conferred by surgical intervention.

**Table 4:** Overall survival and renal cancer-specific survival.

Treatment	No surgery	Surgery
Overall Survival		
N	695	695
1-year survival (95% CI)	23.96% (21.17%–27.12%)	60.97% (57.76%–64.37%)
3-year survival (95% CI)	8.05% (6.41%–10.11%)	41.79% (38.27%–45.64%)
5-year survival (95% CI)	3.56% (2.53%–5.01%)	31.50% (27.95%–35.50%)
Kidney cancer-special survival		
N	3795	3733
1-year survival (95% CI)	28.40% (25.29%–31.90%)	63.87% (60.63%–67.27%)
3-year survival (95% CI)	11.47% (9.31%–14.13%)	46.23% (42.57%–50.21%)
5-year survival (95% CI)	5.77% (4.22%–7.88%)	36.20% (32.36%–40.49%)

CI, Confidence interval.

**Fig 2:**
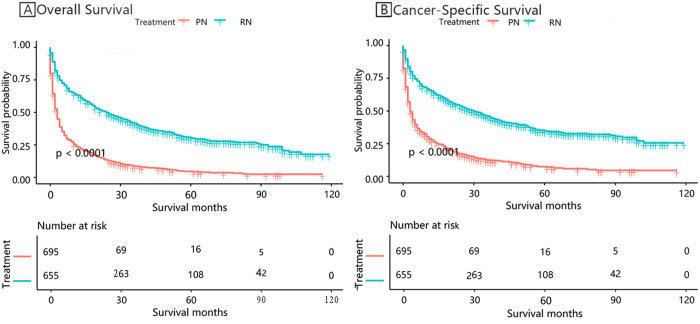
Survival outcomes stratified by treatment modalities.

As illustrated in Table 4 and Fig 2A, patients in the surgery group achieved significantly higher OS compared to their non-surgical counterparts (p < 0.0001). The KM survival curve analysis corroborates these results, providing robust evidence of the survival benefit observed in the tabulated data.

In a similar fashion, RCC-SS, as presented in Table 4 and Fig 2B, was notably higher among patients who underwent surgery compared to those who did not (p < 0.0001). The 1-year, 3-year, and 5-year RCC-SS rates in the surgery group were 63.87%, 46.23%, and 36.20%, respectively, whereas in the non-surgery group, the corresponding rates were 28.40%, 11.47%, and 5.77%. This pattern was further validated by KM survival curve curve analysis, which consistently revealed a marked survival benefit favoring those who received surgical treatment.

**Fig 3:**
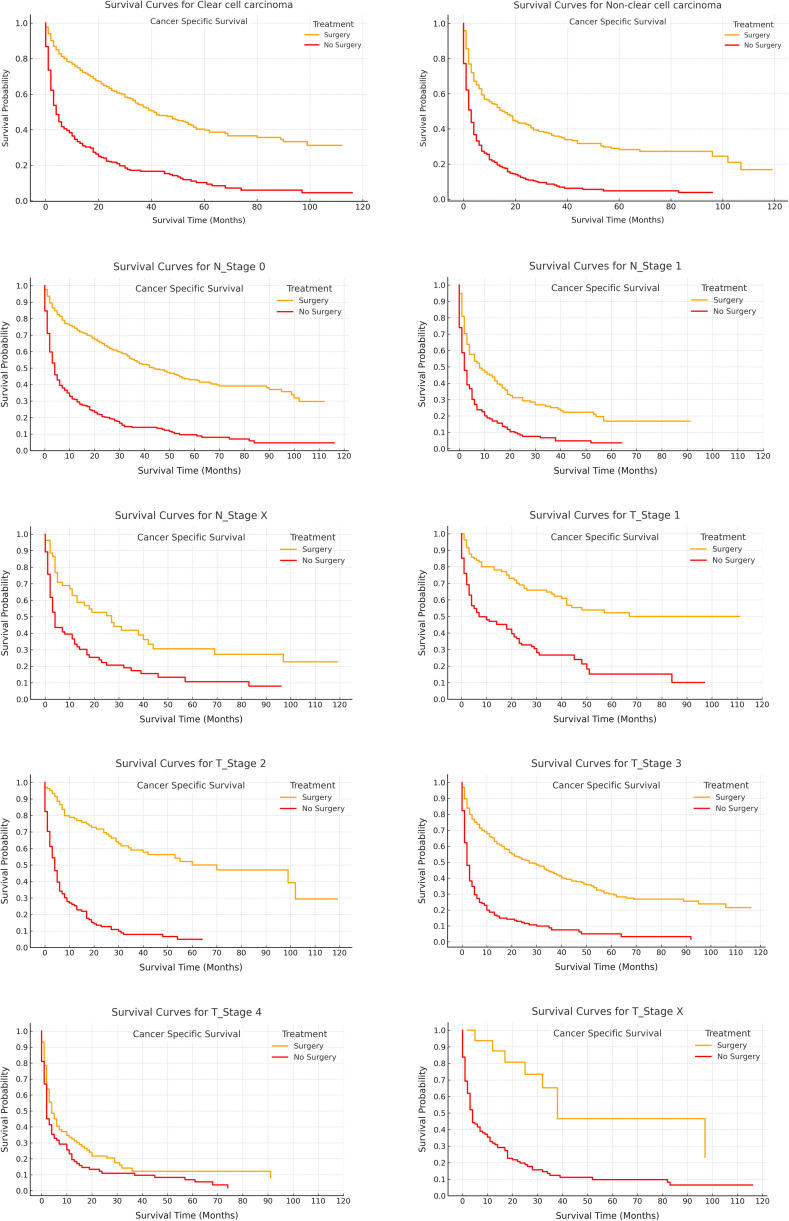
Kidney cancer-specific survival stratified by treatment modalities.

**Fig 4:**
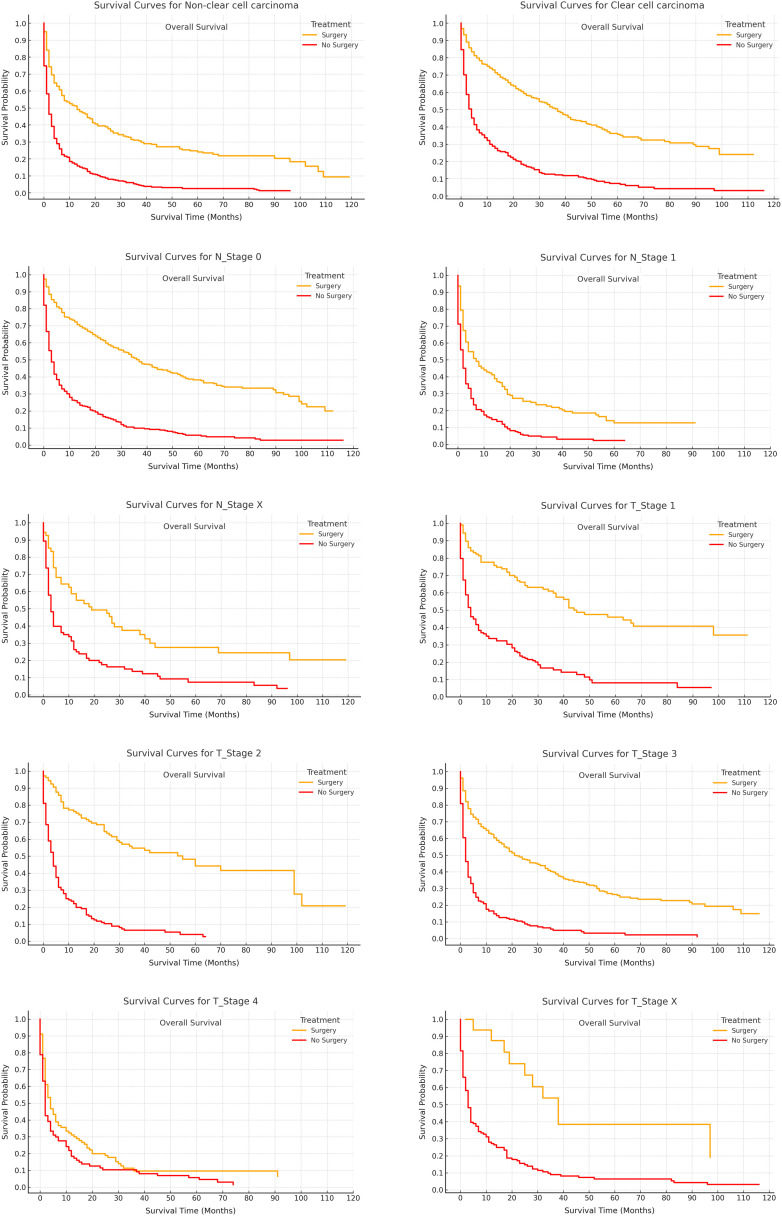
Overall survival stratified by treatment modalities.

Figs 3 and 4 further highlight this survival benefit, with OS and RCC-SS consistently favoring the surgery group across all stratifications. Statistically significant differences were observed (p < 0.05), reaffirming the critical role of surgical intervention in improving both OS and RCC-SS outcomes.

### Analysis of the cumulative incidence of competitive risk

**Fig 5:**
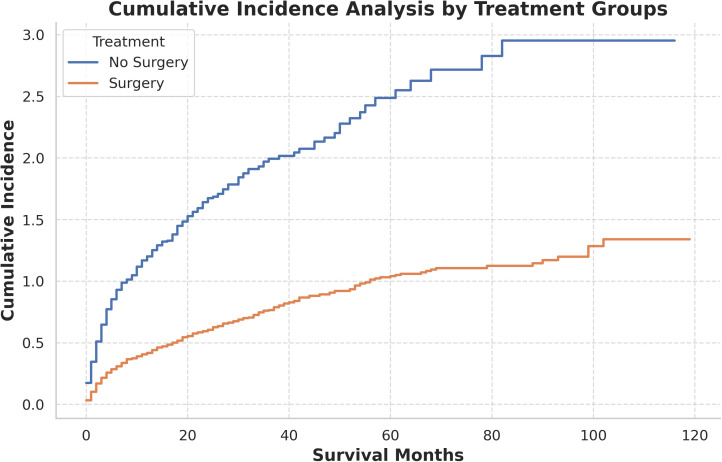
Analysis of the cumulative incidence of competing risks among different treatment modalities.

As illustrated in Fig 5, the cumulative incidence curve for the surgery group ascends more gradually compared to the non-surgery group, suggesting that surgical intervention may mitigate the cumulative incidence of adverse events. The divergence between the two groups becomes increasingly pronounced over time, underscoring the potential protective effect of surgical treatment during long-term follow-up.

**Fig 6:**
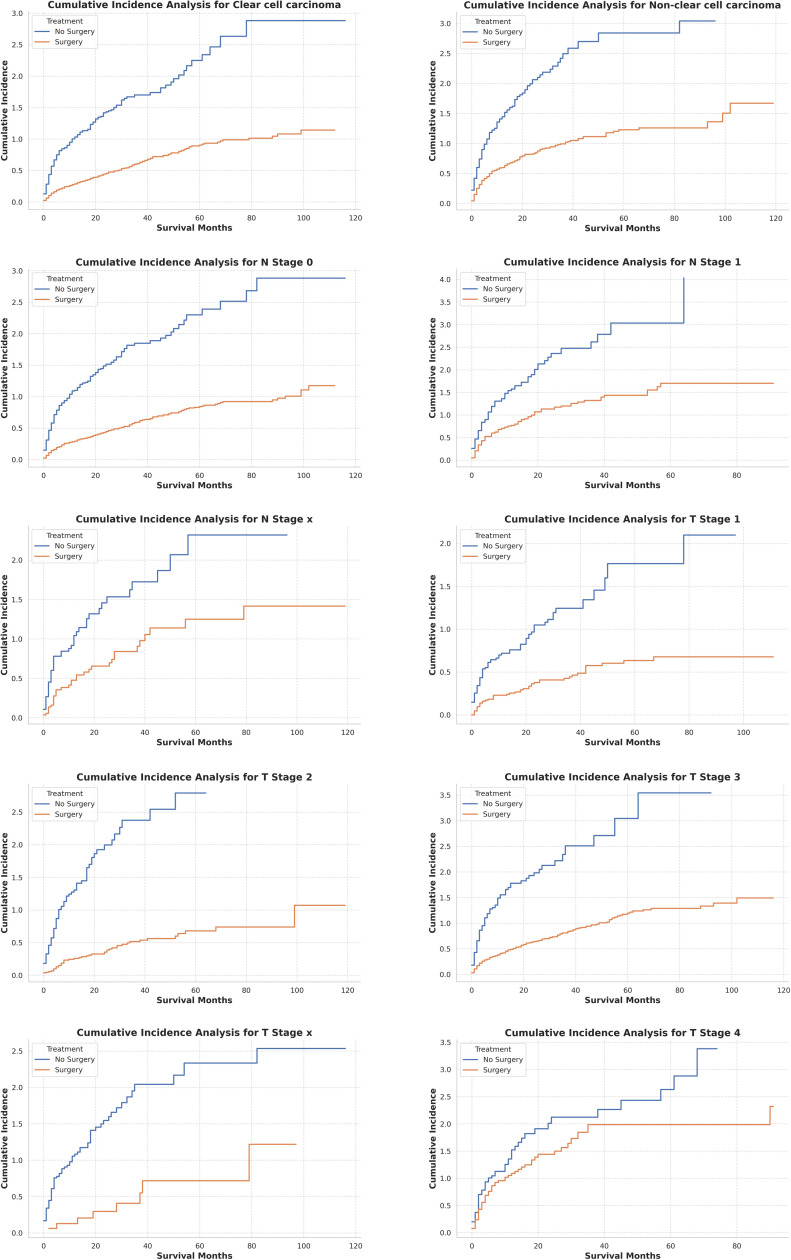
Analysis of the cumulative incidence of competing risks among different subgroups.

Moreover, as illustrated in Fig 6, subgroup analyses examining the cumulative incidence of competing risks across various categories (T1–Tx, N0–Nx, clear cell, and non-clear cell histologies) consistently revealed a more gradual increase in the surgery cohort compared to the non-surgery cohort. This pattern suggests a diminished cumulative incidence of adverse events associated with surgical intervention. The widening gap between the treatment groups over time further underscores the potential long-term protective effects of surgery, spanning across distinct tumor stages, nodal statuses, and histological subtypes.

## Discussion

This study thoroughly assessed the survival advantages of CN in patients with mRCC by utilizing data from the SEER database. By offering real-world, population-based evidence, our results emphasize the critical role of CN in refining treatment approaches for mRCC. The results revealed significant reductions in both overall mortality and RCC-specific mortality, with consistent survival advantages observed across diverse subgroups. These findings validate the integration of CN within modern multimodal therapeutic frameworks, particularly in combination with targeted and immune therapies, while also providing a foundation for future prospective studies to refine its clinical application.

In Fig 2 through 4, Kaplan-Meier survival curves were employed to juxtapose OS and RCC-SS between patients who underwent CN and those who did not. Fig 2 unequivocally demonstrated a marked survival advantage for the surgical cohort, with 1-year, 3-year, and 5-year OS rates of 60.97%, 41.79%, and 31.50%, respectively, in contrast to 23.96%, 8.05%, and 3.56% in the non-surgical group (p < 0.0001). Likewise, Fig 3 further corroborated this observation, revealing superior RCC-SS rates in the surgical group, thereby accentuating the pivotal role of CN in enhancing survival outcomes. Additionally, Fig 4 depicted that the survival benefits were uniformly evident across all clinical subgroups, with particular prominence in patients presenting with high-grade or advanced-stage tumors, thus reaffirming the indispensable role of CN in the management of metastatic renal cell carcinoma.

Furthermore, Figs 5 and 6 focused on competing risks, utilizing cumulative incidence curves to deepen the analysis. Fig 5 revealed that the surgical group experienced a more gradual escalation in competing risks, suggesting that CN not only prolonged survival but also mitigated the occurrence of other adverse events. Over time, the disparity between the surgery and non-surgery groups expanded, underscoring the enduring advantages conferred by CN. In addition, Fig 6 provided a more granular analysis by evaluating clinical subgroups, consistently showing lower competing risks in the surgical cohort, particularly among patients with T1–T3 tumors and clear cell carcinoma. These findings further reinforce CN’s efficacy in diminishing both renal cancer-specific and overall mortality, offering substantial long-term survival benefits, particularly within high-risk populations.

We demonstrated that CN is associated with significantly improved overall survival (OS) and RCC-specific survival RCC-SS in patients with mRCC. By analyzing a large, population-based cohort from the SEER database and employing propensity score matching to minimize selection bias, we found that CN resulted in a 71% reduction in all-cause mortality risk and a 71% reduction in RCC-specific mortality risk compared to non-surgical management. These survival benefits were consistently observed across various subgroups categorized by age, sex, race, tumor grade, histologic subtype, T stage, and N stage. Additionally, Kaplan-Meier survival curves and analyses of cumulative incidence in the context of competing risks further validated that CN not only extends survival but also diminishes the cumulative incidence of RCC-related mortality over time.

Our multivariate analysis further highlighted the magnitude of CN’s impact. CN was associated with a 71% reduction in all-cause mortality (HR = 0.29, 95% CI = 0.25–0.33, p < 0.0001) and a 71% reduction in RCC-specific mortality (HR = 0.29, 95% CI = 0.25–0.34, p < 0.0001). Survival benefits were consistent across multiple subgroups, demonstrating improved 1-, 3-, and 5-year survival rates in the CN group compared to the non-surgical group. For instance, the 5-year OS rate was 31.50% in the CN group versus only 3.56% in the non-surgical group (p < 0.0001). These robust statistical findings substantiate the critical role of CN in appropriately selected patients.

Stratified analysis lends further biological plausibility to these findings. Patients with lower-grade tumors (e.g., G2: HR = 0.17, p < 0.0001) and early-stage tumors (e.g., T1: HR = 0.34, p < 0.0001) derived the greatest survival benefits, likely due to reduced metastatic burden and better surgical tolerance. However, even patients with advanced-stage tumors (e.g., T4: HR = 0.70, p = 0.0291) or high-grade disease showed significant survival improvements, suggesting that CN may attenuate systemic disease progression even in aggressive cases. Additionally, subgroup analyses by demographic factors demonstrated consistent benefits across age, sex, and race, reinforcing the generalizability of these results. Collectively, these findings underscore the rationale for integrating CN into multimodal treatment paradigms, particularly alongside modern systemic therapies.

Moreover, our findings carry significant clinical implications. Cytoreductive nephrectomy (CN) confers notable survival benefits, particularly in younger patients, those with low-grade tumors, or those diagnosed at earlier stages. Remarkably, this advantage persists even in patients with high-grade or advanced-stage tumors. These results emphasize the importance of careful patient selection: CN is most effective in patients with favorable surgical tolerance and limited tumor burden. Additionally, CN should be integrated into a multimodal treatment approach, ideally combined with targeted therapies or immune checkpoint inhibitors, to enhance therapeutic efficacy. Although our study did not include these therapies, existing literature supports their synergistic effects with CN, particularly in high-risk populations. Ultimately, treatment should be personalized based on factors such as age, tumor stage, grade, and overall health. In summary, while CN offers significant survival benefits, its application must be tailored to maximize clinical efficacy.

The survival advantage conferred by CN likely stems from its ability to reduce tumor burden, modulate the host immune microenvironment, and synergize with systemic therapies [3,11,14]. By excising the primary tumor, CN may mitigate the immunosuppressive effects of pro-inflammatory and immunosuppressive factors, such as VEGF and TGF-β [15,16]. Additionally, CN may enhance patient responsiveness to immune checkpoint inhibitors and targeted therapies [17,18]. This benefit is particularly critical in the current era of systemic treatment advances [19–21]. Our study further confirms that CN significantly prolongs survival in specific patient subgroups, such as those with early-stage and low-grade tumors, underscoring its pivotal role within the multimodal treatment framework.

In addition to CN, Stereotactic Body Radiation Therapy (SBRT) is gaining attention in mRCC management, especially for patients unfit for surgery or those with oligometastatic disease [22]. SBRT, a highly precise, non-invasive modality, provides local control when surgery is not feasible [23]. For non-surgical candidates, SBRT can complement CN to effectively reduce tumors, enhancing survival and quality of life [24]. Furthermore, SBRT may synergize with systemic therapies like immune checkpoint inhibitors and targeted therapies to improve outcomes [25]. Integrating SBRT into multimodal treatment, particularly in high-risk or oligometastatic patients, could optimize efficacy where CN alone is insufficient. Future studies should explore the combined potential of SBRT and CN, especially in the context of advancing systemic treatments [22].

Our findings align with and extend previous key studies highlighting the survival benefits of CN in mRCC [26]. A retrospective study demonstrated significantly improved 5-year OS and RCC-SS in mRCC patients undergoing CN (HR = 0.29, *p* < 0.0001), consistent with our multivariate analysis [26]. Similarly, a systematic meta-analysis confirmed that CN combined with targeted therapies significantly enhances survival, reaffirming its role in multimodal treatment strategies [19]. Moreover, the study by Mejean et al. [11] underscored the importance of CN, particularly when integrated with targeted therapies, as a cornerstone of the modern therapeutic framework Consistent with our findings, Massari et al.‘s [27] meta-analysis revealed that patients with low-grade (G2) and low-stage (T1) tumors derive the greatest benefit, likely attributable to better surgical tolerance and reduced tumor burden.

However, the findings of the CARMENA and SURTIME trials diverge from our results, as these randomized controlled studies reported limited survival benefits of CN in high-risk patients, such as those with extensive metastatic burden or poor performance status [11,12]. For example, the SURTIME trial conducted by Bex et al. [12] suggested that CN may not confer significant survival advantages in the context of advanced systemic therapies, indicating that its efficacy might be constrained in these subgroups. These discrepancies emphasize the need for further prospective studies to better delineate the role of CN in diverse clinical contexts.

This study is not without its limitations. The retrospective design and reliance on SEER data inherently introduce potential biases and unmeasured confounders, despite the implementation of propensity score matching. [28]. Additionally, the absence of data regarding the use of TKIs and ICIs within the SEER database curtailed our capacity to fully assess their impact in conjunction with CN on survival outcomes in patients with mRCC [29]. As TKIs and ICIs have become integral to the contemporary management of mRCC, our findings may not fully encapsulate the current therapeutic paradigm. Moreover, the lack of detailed clinical information—such as patient performance status, comorbidities, and metastatic burden—may limit the generalizability of our results to certain patient subgroups [30]. Lastly, the omission of critical variables necessary for the Memorial Sloan Kettering Cancer Center (MSKCC) risk score, including lactate dehydrogenase (LDH), hemoglobin levels, calcium concentration, Karnofsky performance status, and the time from diagnosis to systemic therapy, precluded the ability to perform robust prognostic risk stratification [31,32]. These limitations may undermine the precision and applicability of our findings.

Future research should utilize more comprehensive datasets that include detailed information on the use of TKIs and ICIs, as well as clinical variables like patient performance status, comorbidities, and metastatic burden. Incorporating key parameters for the MSKCC risk score, such as LDH, hemoglobin, calcium levels, Karnofsky performance status, and time to systemic therapy, will enable more accurate prognostic risk stratification. These improvements would provide clearer insights into the synergistic effects of CN and modern therapies in mRCC treatment.

In conclusion, our study provides robust real-world evidence for the survival benefits of CN in mRCC, underscoring its critical role within multimodal treatment frameworks. Addressing current limitations and exploring the synergy between CN and systemic therapies will enable the development of patient-specific strategies, maximizing therapeutic outcomes and advancing the field of mRCC management.

## Conclusion

CN significantly improves survival in mRCC patients, with consistent benefits across subgroups. This study highlights CN’s critical role in multimodal treatment strategies and provides valuable evidence for its integration with systemic therapies, supporting personalized approaches for optimized patient outcomes.

## Supporting information

S1 Table Stratified analysis of the association between treatment method and mortality. (XLSX)

## References

[1] Siegel RL, Miller KD, Fuchs HE, Jemal A. Cancer statistics, 2022. CA Cancer J Clin. 2022;72(1):7–33. 35020204 10.3322/caac.21708

[2] Padala SA, Barsouk A, Thandra KC, Saginala K, Mohammed A, Vakiti A. World J Oncol. 2020;11(3):79–87. 32494314 10.14740/wjon1279 PMC7239575

[3] Choueiri TK, Motzer RJ. Systemic Therapy for Metastatic Renal-Cell Carcinoma. N Engl J Med. 2017;376(4):354–66. doi: 10.1056/NEJMra1601333 28121507

[4] Bianchi M, Sun M, Jeldres C, Shariat SF, Trinh Q-D, Briganti A, et al. Distribution of metastatic sites in renal cell carcinoma: a population-based analysis. Ann Oncol. 2012;23(4):973–80. doi: 10.1093/annonc/mdr362 21890909

[5] Delacroix SE, Chapin BF, Wood CG. Cytoreductive Nephrectomy. In: Lara JPN, Jonasch E, editors. Kidney Cancer: Principles and Practice. Berlin, Heidelberg: Springer Berlin Heidelberg; 2012. p. 109–21.

[6] Napolitano L, Manfredi C, Cirillo L, Fusco GM, Passaro F, Abate M, et al. Cytoreductive Nephrectomy and Metastatic Renal Cell Carcinoma: State of the Art and Future Perspectives. Medicina (Kaunas). 2023;59(4):767. doi: 10.3390/medicina59040767 37109725 PMC10143323

[7] Studentova H, Spisarova M, Kopova A, Zemankova A, Melichar B, Student V, et al. The evolving landscape of cytoreductive nephrectomy in metastatic renal cell carcinoma. Cancers (Basel). 2023;15(15). 10.3390/cancers15153855 PMC10417043 37568671

[8] Park JS, Kim J, Jeon J, Lee J, Jang WS, Lee SH, et al. The role of cytoreductive nephrectomy in metastatic renal cell carcinoma in immune-oncology era (SEVURO-CN): study protocol for a multi-center, prospective, randomized trial. Trials. 2024;25(1):447. doi: 10.1186/s13063-024-08234-2 38961439 PMC11223430

[9] Iisager L, Ahrenfeldt J, Donskov F, Ljungberg B, Bex A, Lund L, et al. Multicenter randomized trial of deferred cytoreductive nephrectomy in synchronous metastatic renal cell carcinoma receiving checkpoint inhibitors: the NORDIC-SUN-Trial. BMC Cancer. 2024;24(1):260. doi: 10.1186/s12885-024-11987-3 38402173 PMC10893632

[10] Zondervan PJ, Bex A. What We Have Learnt from CARMENA and SURTIME and What Should Be Done Differently in Future Trials on Cytoreductive Nephrectomy. Kidney Cancer. 2022;6(2):95–103. doi: 10.3233/kca-220004

[11] Mejean A, Ravaud A, Thezenas S, Colas S, Beauval JB, Bensalah K, et al. Sunitinib Alone or after Nephrectomy in Metastatic Renal-Cell Carcinoma. N Engl J Med. 2018;379(5):417–27. 29860937 10.1056/NEJMoa1803675

[12] Bex A, Mulders P, Jewett M, Wagstaff J, van Thienen JV, Blank CU, et al. Comparison of Immediate vs Deferred Cytoreductive Nephrectomy in Patients With Synchronous Metastatic Renal Cell Carcinoma Receiving Sunitinib: The SURTIME Randomized Clinical Trial. JAMA Oncol. 2019;5(2):164–70. doi: 10.1001/jamaoncol.2018.5543 30543350 PMC6439568

[13] Van Praet C, Slots C, Vasdev N, Rottey S, Fonteyne V, Andras I, et al. Current role of cytoreductive nephrectomy in metastatic renal cell carcinoma. Turk J Urol. 2021;47(Supp1):S79–84. 10.5152/tud.2021.21006 PMC8057358 35929921

[14] Bakouny Z, El Zarif T, Dudani S, Connor Wells J, Gan CL, Donskov F, et al. Upfront Cytoreductive Nephrectomy for Metastatic Renal Cell Carcinoma Treated with Immune Checkpoint Inhibitors or Targeted Therapy: An Observational Study from the International Metastatic Renal Cell Carcinoma Database Consortium. Eur Urol. 2023;83(2):145–51. doi: 10.1016/j.eururo.2022.10.004 36272943

[15] Gabrilovich DI, Nagaraj S. Myeloid-derived suppressor cells as regulators of the immune system. Nat Rev Immunol. 2009;9(3):162–74. doi: 10.1038/nri2506 19197294 PMC2828349

[16] Motzer RJ, Jonasch E, Boyle S, Carlo MI, Manley B, Agarwal N, et al. NCCN Guidelines Insights: Kidney Cancer, Version 1.2021. J Natl Compr Canc Netw. 2020;18(9):1160–70. 32886895 10.6004/jnccn.2020.0043 PMC10191771

[17] Barata PC, Rini BI. Treatment of renal cell carcinoma: Current status and future directions. CA Cancer J Clin. 2017;67(6):507–24. doi: 10.3322/caac.21411 28961310

[18] Li C, Wang R, Ma W, Liu S, Yao X. Do Metastatic Kidney Cancer Patients Benefit From Cytoreductive Nephrectomy? A Real-World Retrospective Study From the SEER Database. Front Surg. 2021;8:716455. doi: 10.3389/fsurg.2021.716455 34557516 PMC8454406

[19] Esagian SM, Ziogas IA, Kosmidis D, Hossain MD, Tannir NM, Msaouel P. Long-Term Survival Outcomes of Cytoreductive Nephrectomy Combined with Targeted Therapy for Metastatic Renal Cell Carcinoma: A Systematic Review and Individual Patient Data Meta-Analysis. Cancers. 2021;13(4). 10.3390/cancers13040695 PMC7915816 33572149

[20] Ishihara H, Omae K, Nemoto Y, Ishiyama R, Tachibana H, Nishimura K, et al. First-line dual immune checkpoint inhibitor therapies versus combination therapies comprising immune checkpoint inhibitors and tyrosine kinase inhibitors for advanced renal cell carcinoma: a comparative analysis of the effectiveness using real-world data. Int J Clin Oncol. 2024;29(4):473–80. 38345708 10.1007/s10147-024-02471-w

[21] Niewada M, Macioch T, Konarska M, Mela A, Goszczyński A, Przekopińska B, et al. Immune checkpoint inhibitors combined with tyrosine kinase inhibitors or immunotherapy for treatment-naïve metastatic clear-cell renal cell carcinoma-A network meta-analysis. Focus on cabozantinib combined with nivolumab. Front Pharmacol. 2023;13:1063178. doi: 10.3389/fphar.2022.1063178 36937206 PMC10020696

[22] Ali M, Mooi J, Lawrentschuk N, McKay RR, Hannan R, Lo SS, et al. The Role of Stereotactic Ablative Body Radiotherapy in Renal Cell Carcinoma. Eur Urol. 2022;82(6):613–22. doi: 10.1016/j.eururo.2022.06.017 35843777

[23] Wang CJ, Christie A, Lin M-H, Jung M, Weix D, Huelsmann L, et al. Safety and Efficacy of Stereotactic Ablative Radiation Therapy for Renal Cell Carcinoma Extracranial Metastases. Int J Radiat Oncol Biol Phys. 2017;98(1):91–100. doi: 10.1016/j.ijrobp.2017.01.032 28587057 PMC5555369

[24] Siva S, Kothari G, Muacevic A, Louie AV, Slotman BJ, Teh BS, et al. Radiotherapy for renal cell carcinoma: renaissance of an overlooked approach. Nat Rev Urol. 2017;14(9):549–63. doi: 10.1038/nrurol.2017.87 28631740

[25] Roh W, Chen P-L, Reuben A, Spencer CN, Prieto PA, Miller JP, et al. Integrated molecular analysis of tumor biopsies on sequential CTLA-4 and PD-1 blockade reveals markers of response and resistance. Sci Transl Med. 2017;9(379):eaah3560. doi: 10.1126/scitranslmed.aah3560 28251903 PMC5819607

[26] Zhao Z, Wu W, Duan X, Zeng G, Liu Y. The value of cytoreductive nephrectomy on the survival of metastatic renal carcinoma patients based on the number of site-specific metastases. PLoS One. 2019;14(4):e0215861. doi: 10.1371/journal.pone.0215861 31013336 PMC6478335

[27] Massari F, Di Nunno V, Gatto L, Santoni M, Schiavina R, Cosmai L, et al. Should CARMENA Really Change our Attitude Towards Cytoreductive Nephrectomy in Metastatic Renal Cell Carcinoma? A Systematic Review and Meta-Analysis Evaluating Cytoreductive Nephrectomy in the Era of Targeted Therapy. Target Oncol. 2018;13(6):705–14. doi: 10.1007/s11523-018-0601-2 30324488

[28] Marchioni M, Bandini M, Preisser F, Tian Z, Kapoor A, Cindolo L, et al. Survival after Cytoreductive Nephrectomy in Metastatic Non-clear Cell Renal Cell Carcinoma Patients: A Population-based Study. Eur Urol Focus. 2019;5(3):488–96. doi: 10.1016/j.euf.2017.11.012 29229582

[29] Marchioni M, Bandini M, Pompe RS, Tian Z, Martel T, Kapoor A, et al. Survival of metastatic renal cell carcinoma patients continues to improve over time, even in targeted therapy era. Int Urol Nephrol. 2017;49(12):2143–9. doi: 10.1007/s11255-017-1703-y 28932952

[30] Yang DX, Khera R, Miccio JA, Jairam V, Chang E, Yu JB, et al. Prevalence of Missing Data in the National Cancer Database and Association With Overall Survival. JAMA Netw Open. 2021;4(3):e211793. doi: 10.1001/jamanetworkopen.2021.1793 33755165 PMC7988369

[31] Fiala O, Finek J, Poprach A, Melichar B, Kopecký J, Zemanova M. Outcomes according to MSKCC risk score with focus on the intermediate-risk group in metastatic renal cell carcinoma patients treated with first-line sunitinib: A retrospective analysis of 2390 patients. Cancers. 2020;12(4). 10.3390/cancers12040808 PMC7225945 32230921

[32] Motzer RJ, Mazumdar M, Bacik J, Berg W, Amsterdam A, Ferrara J. Survival and prognostic stratification of 670 patients with advanced renal cell carcinoma. J Clin Oncol. 1999;17(8):2530–40. doi: 10.1200/JCO.1999.17.8.2530 10561319

